# Generative tissue modeling for customized biomechanical analysis: a data-driven synthesis framework of simulation-ready 3D shapes

**DOI:** 10.3389/fbioe.2026.1761981

**Published:** 2026-04-07

**Authors:** Junmin Ma, Zhi Wang, Zuxing Wu, Jie Li, Zhouquan Lv, Taisong Cui, Biwei Zhou, Yuanshuai Chen, Yuan Huang, Jisi Tang

**Affiliations:** 1 State Key Laboratory of Intelligent Vehicle Safety Technology, Chongqing, China; 2 Key Laboratory of Biorheological Science and Technology of Ministry of Education, Chongqing University, Chongqing, China; 3 Bioengineering College, Chongqing University, Chongqing, China; 4 International Center for Automotive Medicine (ICAM), University of Michigan, Ann Arbor, MI, United States

**Keywords:** 3D generative model, biomechanics, non-rigid registration, shape analysis, tissue modeling

## Abstract

**Introduction:**

Conventional computational modeling of human body and tissue relies on time-consuming reconstruction from medical images. Instead, generative artificial intelligence synthesizes novel samples with few marginal costs. However, existing models are incompatible with 3D data structures. This study aims to establish a 3D generative tissue modeling pipeline. The key objective is to address the following technical bottlenecks: 1) tackle the neural-network incompatibility of discretization-agnostic meshes, 2) formulate a sequence-type architecture for shape prior learning and generation, and 3) realize smooth source-to-target mapping.

**Methods:**

We propose a data-driven non-rigid registration that learns geometry-informed features through self-supervised pretraining, and infers high-fidelity correspondences accordingly. A template mesh is uniformly registered onto 90 femur shapes to align inter-vertex connectivity. A variational autoencoder (VAE) is trained on the structurally aligned samples, and synthesizes novel shapes thereafter. Left femur of a baseline finite-element model is mapped onto the synthesized instances respectively, and the femurs are computationally loaded to investigate biomechanics with diverse morphologies.

**Results:**

Tuning the six latent dimensions of the trained VAE independently generates shapes in heterogenous morphological patterns, i.e., overall size, overall bending curvature, slenderness, shaft length, fine-level shaft curvature, and local epiphysis/metaphysis style. Quantitatively, varying the first latent results in an 83.4 mm change in femur length, while the second latent controls the equivalent radius of shaft segment in a range from 66.8 mm to 862.6 mm. The VAE model synthesizes geometrically valid shapes within up to 3 standard deviations (>99.7%) of the entire latent. 10 new femurs are synthesized and registered onto a baseline finite-element model in less than 100 s for each case. Preliminary analysis with three-point bending load reveals morphological variation might have a significant influence on deformation pattern and bending biomechanics.

**Conclusion:**

This study establishes a contemporary generative paradigm for tissue modeling, and demonstrates efficacy and feasibility of biomechanics investigation with synthetic shapes. Our method produces high-fidelity, simulation-ready models in only minutes. The pretraining scheme is scalable to multiple anatomical structures and sheds light to foundation model of 3D anatomies, which might promisingly benefit a lot of production workflows, e.g., active-passive vehicle safety, robot-assisted surgery, and all biomechanics/morphology-relevant tasks.

## 1 Introduction

Understanding the impact of different human skeletal morphologies on biomechanical responses is crucial to fields such as injury biomechanics, orthopedic prosthesis design, and vehicle safety (Hu et al., 2019). Currently, Finite Element Human Models (FE-HBMs), e.g., the Total Human Model for Safety (THUMS) (Iwamoto et al., 2002) and the Global Human Body Model Consortium (GHBMC) (Hayashi et al., 2008), have been widely used for biomechanical analysis. High-fidelity simulation with HBMs provides a cost- and time-efficient yet biofidelic way in addressing biomechanics-centered tasks, especially in automotive safety field. However, in this domain, different human characteristics, e.g., height and weight, exhibit considerable variations in crash injury responses (Carter et al., 2014). Therefore, constructing a statistical, parametric model that represents diverse human body shapes is of great importance. Conventionally, most numerical human modeling methods heavily rely on medical imaging techniques, reconstructing 3D human models from CT or MRI data. However, this case-by-case approach incurs linear computational costs, making it both expensive and time-consuming. More critically, such “one-at-a-time” reconstruction is not generative, rendering the resulting models highly subject-specific and thus lacking the generalizability required for large-scale populational or demographic analysis.

To address these limitations, researchers have increasingly focused on Statistical Shape Models (SSMs), a framework inherently suited for anthropometry and demographics. Typically constituted by Principal Component Analysis (PCA) results and regression models, SSMs enable the generation or synthesis of new body or tissue samples, such as ribs (Shi et al., 2014), femurs (Klein et al., 2015), and whole-body models (Zhang et al., 2017). When combined with mesh morphing techniques, the synthetic shapes can straightforwardly benefit biomechanics studies across population in vehicle crashes (Hu et al., 2019). However, the shape space in classic SSMs is inherently spanned by a linear basis. While effective for global variance, this linear superposition often faces challenges in compactly representing high-dimensional morphological details; capturing fine-level, coupled deformations typically necessitates retaining a significantly larger number of principal components. Furthermore, the boundaries of the valid subspace in PCA are often ill-defined. Optimization within this unbounded space can lead to unrealistic geometric artifacts, necessitating additional regularization terms (e.g., L2 penalties) that may inadvertently compromise the fidelity of the target dimensions.

Beyond these geometric constraints, the standard regression-based approach limits the model’s editability. By simply regressing PC scores on specific parameters of interest (e.g., age, height). This approach limits the model’s ability to handle free-form conditional generation for unconsidered characteristics, lacking the editability and flexibility required for advanced industrial practices. From a workflow perspective, conventional SSMs are neither strictly consistent nor sufficiently automatic. They often necessitate manual or semi-automatic registration steps to align sample meshes prior to analysis, introducing potential human error and hindering scalability. While template-based corrections are manageable for a single bone or organ, the workload increases exponentially when modeling integrated systems such as the body exterior, skeletons, and internal organs simultaneously. In such high-dimensional scenarios, maintaining consistent point-to-point correspondence across hundreds of disparate landmarks becomes a prohibitive manual bottleneck. Furthermore, the fidelity of a generative model is fundamentally rooted in the quality of training data. Even minor inconsistencies in manual annotations during the alignments of complex anatomical assemblies can introduce noise into the learned prior distribution.

With the rapid advancement of deep learning, contemporary artificial intelligence (AI) technologies are reshaping the research paradigm of computational biomechanics. In particular, the emergence of Generative AI has enabled researchers to learn the latent distributions of shape data and generate three-dimensional anatomical structures with high anatomical plausibility. Unlike traditional models, generative models, e.g., Variational Autoencoders (VAEs) (Kingma and Welling, 2013), Generative Adversarial Networks (GANs) (Goodfellow et al., 2020), and Diffusion Models (Sohl-Dickstein et al., 2015), can capture complex morphological variations within nonlinear latent spaces, thereby producing diverse and controllable virtual individuals while preserving geometric fidelity.

However, the practical application of generative models in finite element analysis still faces critical technical challenges. Most current 3D generative approaches rely on implicit representation methods, such as NeRF (Mildenhall et al., 2021) and 3D Gaussian (Kerbl et al., 2023) representations. While these methods leverage continuous field functions and architectures like 3D U-Net (Çiçek et al., 2016) to achieve efficient, photorealistic high-dimensional synthesis, they fundamentally lack constraints on explicit topological structures. Consequently, although the generated outputs may appear visually realistic, they are essentially visual reconstructions lacking explicit topological definition, rather than engineered models. Specifically, implicit reconstructions are rarely “production-ready”—defined here as possessing not only anatomical visual accuracy but also realistic biomechanical responsiveness under physiological loads. Furthermore, they fail to be “simulation-ready”, which requires a watertight volumetric mesh structure with high element quality (e.g., no holes) capable of being directly employed in finite element solvers without manual repair.

This limitation highlights a pressing need for generative frameworks based on explicit representations, which can inherently guarantee the mesh quality and topological consistency required for large-scale biomechanical analysis. While explicit representations, e.g., meshes, are more intuitive and computationally efficient than their implicit counterparts, integrating them with deep learning poses unique challenges. Typically, explicit representations discretize space in a structure-agnostic manner, which results in poor compatibility with conventional neural network architectures. For example, a group of 3D surfaces could be tessellated into arbitrary triangulations, which makes it impractical to learn the global prior with a uniformly predefined network.

Concerning the urgent need and challenges above, this study aims to establish a fully data-driven generative framework. Our motivation emerges from the insight that constructing the prior is as important as dimension reduction, because the prior essentially defines the boundary that encapsulates valid subjects. Since compressing nontrivial and fine-level details demands a large number of multiscale bases under a linear framework, the current study concentrates on prior learning through neural networks which are naturally nonlinear from the architectural perspective.

The specific objectives are as follows: 1) To resolve the incompatibility between mesh representations and neural networks caused by discretization-agnostic characteristics, and thereby overcome the long-standing limitation on large-scale pretraining for anatomical modeling. 2) To learn the shape prior distribution within a nonlinear, compact and informative latent space. By learning a more expressive space, the framework ensures that the generated simulation-ready meshes are more anatomically accurate for downstream high-fidelity FEA. 3) To eliminate geometric inconsistencies among non-rigid anatomical shapes. This process aligns the inconsistent mesh data and geometrically preserves the underlying structural topology. Furthermore, it benefits the simultaneous encoding of multiscale spatial features across a wide frequency spectrum. This allows accurate and smooth pattern transfer from baseline finite element models to newly generated shapes, and thus ensuring their suitability for biomechanical simulation.

## 2 Materials and methods

### 2.1 Overview

**FIGURE 1:**
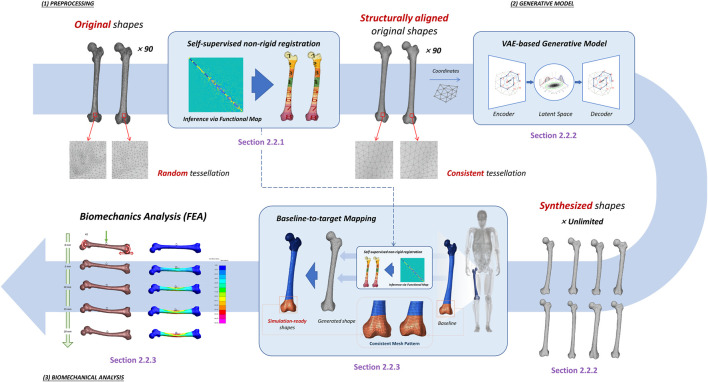
Overview of the proposed methodological workflow. The pipeline establishes a complete framework from raw imaging data to biomechanical analysis. (1) Preprocessing (Section 2.2.1): Segmented raw femur shapes are structurally aligned *via* a self-supervised non-rigid registration approach that unifies the mesh connectivity across original randomly tessellated samples. (2) Generative Model (Section 2.2.2): A variational autoencoder is pretrained on the registered dataset to encode the femur shape prior into a high-dimensional Gaussian distribution, and novel samples are synthesized through the trained VAE. (3) Biomechanical Analysis (Section 2.2.3): Non-rigid registration module is leveraged again to align a baseline finite-element femur model (e.g., from THUMS). onto the synthesized shapes. The morphology-various samples are subject to three-point bending load through finite-element analysis, and the biomechanical responses are investigated.

We propose a data-driven pipeline that learns stochastic 3D shape prior distribution from the explicitly represented shapes and formulates a generation-based workflow that synthesizes production-ready samples. As illustrated in Figure 1, the pipeline proceeds through the following steps: 1. High-fidelity non-rigid registration infers geometric correspondence across heterogeneous meshes; 2. Shape prior learning by variational autoencoder; 3. Generate new shapes, and synthesize simulation-ready subjects accordingly; and 4. Finite-element analysis with the synthesized shapes to investigate their biomechanical responses respectively. This study addresses the challenges in learning shape distributions from discretization-agnostic representations, and demonstrates end-to-end workflow that joins syn-thesis and biomechanics analysis.

### 2.2 Method

#### 2.2.1 3D non-rigid registration

To learn generative shape priors from discretization-agnostic segmentations, we first establish high-fidelity correspondence across the heterogeneous shapes.

1) Self-supervised 3D representation learning

**FIGURE 2:**
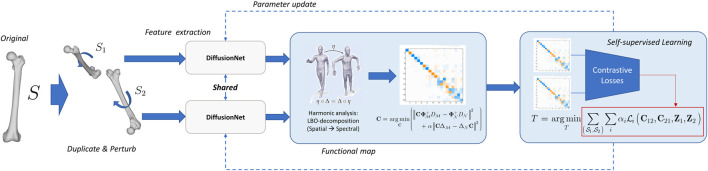
Overview of the self-supervised representation learning pipeline. A Siamese network composed by DiffusionNet is established to learn discretization- and affine-transformation-invariant shape features through contrastive alignment losses in spectral space.

Given a cohort of surfaces $\left\{S_{i}\right\}$ where i=1,...,N representing recruited bone shapes (e.g., femurs), we seek a transformation T that extracts geometrically meaningful embeddings, i.e., deep shape representations, which are discretization-invariant. The self-supervised pretraining workflow is shown in Figure 2.

A Siamese network is built with two branches dealing with randomly perturbed paired instances $S_{1},S_{2}$ of the same input shape respectively. Each copy undergoes random rotation $R\in SO\left(3\right)$ and isotropic scaling $s∼U\left(0.9,1.1\right)$ . The vertex coordinates of the perturbed surfaces $X_{1},X_{2}\in R^{n\times 3}$ serve as input to the backbone, i.e., DiffusionNet (Sharp et al., 2022), with the transformations denoted as T . We selected DiffusionNet as the feature extractor because it enables discretization-agnostic learning by simulating heat diffusion processes on manifolds. Unlike traditional Graph Neural Networks that rely on fixed vertex and edge connectivity, this architecture utilizes the Laplace-Beltrami Operator to emulate diffusion-driven message flow. Consequently, the network becomes inherently tessellation-invariant. This characteristic is critical as anatomical structures reconstructed from medical images are usually represented by agnostic triangulation patterns. Furthermore, the heat diffusion-based message passing scheme allows the model to learn geometry-informed features that are robust to near-isometric perturbations. Such robustness is essential for establishing high-fidelity correspondences during the non-rigid registration phase where most instances within the same category are roughly alike from a geometric perspective.

The two branches share the same weights. The output embeddings $F_{1},F_{2}$ are projected onto the spectral domain using the truncated eigenfunctions of the Laplace-Beltrami Operator (LBO), achieving spectral features $Z_{i}=\Phi_{i}^{+}TX_{i}$ , where $\Phi_{i}^{+}$ denotes the Moore-Penrose pseudoinverse of the truncated bases computed on $S_{i}$ . $Z_{i}$ is naturally discretization-invariant. To measure spectral consistency between the two perturbed copies, we adopt the notion of functional maps (Ovsjanikov et al., 2012). Briefly, a functional map encodes shape-to-shape correspondence as a compact linear operator $C_{12}:L^{2}\left(S_{1}\right)\to L^{2}\left(S_{2}\right)$ , represented in the low-frequency basis of the Laplace–Beltrami operator by a small matrix $C_{12}\in R^{k\times k}$ . Thereafter, the backbone network is optimized to enforce spectral consistency and information richness of the features *via* a group of loss items computed between $Z_{1}$ and $Z_{2}$ , as formulated in Equation 1

$$T=\underset{T}{\mathrm{argmin}}\sum_{\left(S_{1},S_{2}\right)}\sum_{i}\alpha_{i}L_{i}\left(C_{12},C_{21},Z_{1},Z_{2}\right),$$ (1)

where $C_{12}$ is the functional map (Ovsjanikov et al., 2012) from $S_{1}$ to $S_{2}$ and *vice versa*, $L_{i}$ loops from i=1 to three denoting the spectral identity loss ( $L_{id}$ , Equation 2), spectral regularization loss ( $L_{reg}$ , Equation 3) and spectral normalization loss ( $L_{norm}$ , Equation 4) respectively, as below:

$$L_{id}={\left\|C_{12}-I\right\|}^{2}+{\left\|C_{21}-I\right\|}^{2}$$ (2)

$$L_{reg}=\sum_{i\in \left(S_{1},S_{2}\right)}\sum_{d\in D}{\left\|{\left(E_{i}\right)}_{d}-{\left({\bar{E}}_{i}\right)}_{d}\right\|}^{1},E_{i}=\left|Z_{i}\right|$$ (3)

$$L_{norm}=\sum_{i\in \left(S_{1},S_{2}\right)}\sum_{d\in D}{\left|{\left\|{\left(E_{i}\right)}_{d}\right\|}^{2}-1\right|}^{1},E_{i}=\left|Z_{i}\right|$$ (4)

where $E_{i}$ is the absolute form of the spectral representation of embedding associated with $S_{1}$ and $S_{2}$ , ${\bar{E}}_{i}$ is an average per channel, D is the total number of channels inside $E_{i}$ . The entire loss is the weighted sum of the incorporated items, those promote perturbation invariance, uniform spectral energy distribution across frequencies, and numerical stability during training respectively.

2) Hybrid correspondence inference

A multistep inference framework that jointly leverages spectral and spatial tools is proposed to infer pointwise correspondences with the learned features.

Again, consider two given shapes, M,N to register, with precomputed LBO eigenfunctions and learned features $F_{i}=TX_{i}$ . The spectral projections are then $Z_{i}=\Phi_{i}^{+}TX_{i}$ , where i=M,N . The functional maps $C_{MN}$ and $C_{NM}$ could be computed following (Ovsjanikov et al., 2012).

Deblurring algorithm (Ezuz et al., 2017) converts the functional map to vertex-to-point map in spatial domain as below, where $P_{NM}$ maps the vertex on M to a general point on N respectively, as formulated in Equation 5:

$$P_{NM}=\underset{P_{NM}}{\mathrm{argmin}}\left({\left\|\Phi_{M}C_{NM}-P_{NM}\Phi_{N}\right\|}^{2}\right)$$ (5)

The functional map essentially acts as a low-pass filter because the transfer matrices are established on truncated spectral bases. Consequently, they lack the resolution to describe fine-level spatial correspondences. This truncation often manifests as artifacts or irregular deformations in the resulting pattern. To recover high-frequency details suppressed by spectral truncation and ensure smooth, anatomically plausible maps, we refine $\tilde{M}$ , i.e., the map of M on N , using an objective inspired by conformal geometry, as formulated in Equation 6:

$$X_{\tilde{M}}=\underset{X_{\tilde{M}}}{\mathrm{argmin}}\left(\begin{array}{l} \alpha_{\theta }\text{mse}\left(\theta_{M},\theta_{\tilde{M}}\right)+\\ \alpha_{tr}\sum_{i\in V_{\tilde{M}}}\left\|D{\left(L_{M}X_{\tilde{M}}\right)}_{i}\right\| \end{array}\right)$$ (6)

where $\theta_{*}$ are angles between adjacent edges in corresponding tangent space, $L_{M}$ is the Laplacian matrix of M , and $D\left(*\right)$ are the tangent residues that preserve only the components orthogonal to the vertex normals, as formulated in Equation 7:

$$D{\left(*\right)}_{i}={\left(*\right)}_{i}-\langle {\left(*\right)}_{i},{\tilde{n}}_{i}\rangle \cdot {\tilde{n}}_{i}$$ (7)

The optimization in (1) recovers the missing fine-level details by promoting conformality of $\tilde{M}$ towards M , and facilitates the map to be smoother.

#### 2.2.2 Generative model

1) Backbone

**FIGURE 3:**
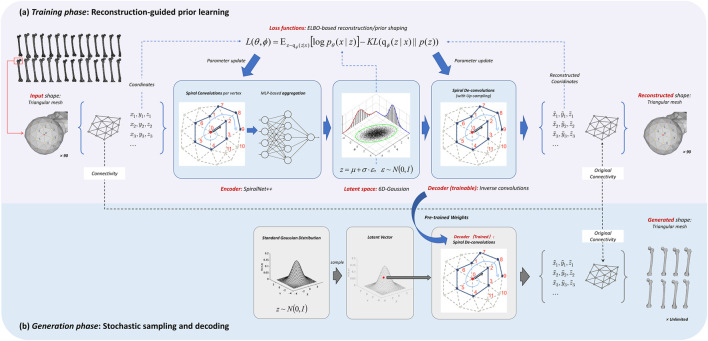
Schematic overview of the Variational Autoencoder (VAE) framework for 3D femur generation. **(a)** *Training phase*: The primary objective is to learn the network weights of the SpiralNet++ encoder and decoder by minimizing the Evidence Lower Bound (ELBO) loss. The input meshes are decomposed into geometric coordinates and topological information. The mesh connectivity (topology) is pre-aligned through global registration and serves as a constant variable throughout the entire training and generation stages, which facilitates graph convolutions. **(b)** *Generation phase*: The shape prior has been compressed into a standard Gaussian distribution in pretraining. It is then ready to sample a latent vector from the stochastic distribution ( $z∼N\left(0,I\right)$ ), and reconstruct the encoded coordinate arrays *via* the trained decoder. The coordinates and constant connectivity map are concatenated to synthesize novel valid, simulation-ready 3D meshes.

The input data for this study consists of 3D triangular meshes with unified topology, primarily represented by a vertex list and a face list. The vertices document 3D spatial coordinates of each point, while the faces encode the inter-vertex connectivity. Although the input vertex coordinates form a fixed-size array (1083 × 3) and reconcile with conventional convolutional operations, e.g., CNN, such approaches neglect the underlying connectivity. We use SpiralNet++ (Gong et al., 2019) as the backbone model for this task, which is designed to handle triangular mesh data with fixed topology through graph-based convolution. The basic idea of SpiralNet++ lies in its use of spiral sampling, which orders the neighboring vertices of each mesh node into a fixed-length sequence based on a predefined spiral traversal pattern. This allows for structured feature aggregation, enabling the network to capture rich geometric representations. Such aggregation not only encodes the local characteristics of individual nodes but also reflects broader contextual geometric relationships, making it particularly effective for modeling anatomical features such as skeletal structures. The detailed reconstruction pipeline is illustrated in Figure 3.

2) Variational autoencoder

Variational Autoencoder (VAE) (Kingma and Welling, 2013) is an update of classical autoencoder. VAE compresses the input data into a distribution over the latent space, outputting a probabilistic representation of the latent variables.

The “variational” aspect of VAE stems from variational inference, which aims to approximate the true posterior distribution by optimizing the Evidence Lower Bound (ELBO). ELBO consists of two key terms: the reconstruction loss and the Kullback-Leibler (KL) divergence. The reconstruction loss measures how accurately the decoder can reconstruct the input data, guiding it to generate outputs consistent with the original input. In our implementation, we adopt the L1 loss to quantify this error, as defined in (Equation 8).

$$L_{recon}=\frac{1}{N}\sum_{i=1}^{N}\left|x_{i}-\hat{x_{i}}\right|$$ (8)

where $x_{i}$ is the original sample, $\hat{x_{i}}$ is the reconstructed output, and N is the total number of samples. The KL divergence measures the distance between the latent distribution produced by the encoder and a predefined prior distribution, usually a standard normal distribution. KL divergence can be represented as (9) for Gaussian-type latent, which is intuitively differentiable *via* backpropagation, as formulated in Equation 9.

$$\text{KL}=\frac{1}{2}\sum_{i=1}^{d}\left(\mu_{i}^{2}+\sigma_{i}^{2}-\log\sigma_{i}^{2}-1\right)$$ (9)

Due to the probabilistic nature of VAE modeling, the latent space tends to be smoother and more continuous.

In this study, the optimization objective is defined as a weighted, as formulated in Equation 1 sum of the reconstruction loss and the KL divergence term (Equation 10), where β and $\lambda_{KL}$ serve as scaling factors to balance geometric accuracy and latent space regularity. This formulation, inspired by the β -VAE (Higgins et al., 2017) paradigm, enables the model to capture high-fidelity morphological details while facilitating controllable generation through a structured latent space.

$$L_{VAE}=\beta \cdot L_{recon}+\lambda_{KL}\cdot D_{KL}\left(q_{\varphi }\left(z|x\right)\left\|p\left(z\right)\right.\right)$$ (10)

#### 2.2.3 Numerical simulation

The autoregressively pretrained VAE encodes the coordinate-based shape prior distribution. It synthesizes new femur shapes through decoding the sampled latent vector. These decoded instances consist of ordered coordinate sequences (vertices), which are inherently solver-agnostic and compatible with standard FEA solvers (e.g., LS-Dyna, ANSYS, and Abaqus). Complete 3D meshes are reconstructed by mapping these coordinates onto the fixed template connectivity.

The left femur of THUMS (Iwamoto et al., 2002) is registered onto the generated shapes. This process transfers the baseline mesh patterns *via* a smooth deformation field. This approach ensures high geometric fidelity by preserving intricate anatomical details, such as the curvature of the greater trochanter. Furthermore, it maintains the rigorous mesh quality required for robust finite element analysis. The registered samples are subject to a three-point bending load, wherein the condyles are constrained with only in-plane rotation available (the longitudinal displacement at distal end is allowed as well). A cylindrical impactor transversely runs into the middle shaft with a quasi-static severity. Deformation of the samples and force-displace history of the impactors are documented and demonstrated to verify if the generated instances are production-ready.

### 2.3 Dataset and implementation

#### 2.3.1 Dataset

The femur shapes used in this study are segmented from previously collected clinical CT scans. The scans are collected in (Du et al., 2018), through a protocol approved by an institutional review board at Tsinghua University (Beijing, China). There are 90 high-quality femur meshes in total. See (Du et al., 2018) for more details on characteristics of the subjects.

In representation learning and registration, the meshes are orientationally aligned by Generalized Procrustes Alignment. A series of preprocessing steps, i.e., topology simplification and vertex redistributions, are carried out to align the data structure. Computations are performed in Polygon Mesh Library (PMP-library) (Daniel and Mario, 2019) and PyTorch3D (Johnson et al., 2020).

A triangular mesh template with 1,083 vertices and 2,162 faces is registered onto every mesh respectively, formulating a new dataset of both geometrically and structurally consistent femur shapes, which are split into 80 samples for training and 10 samples for testing in the generative model establishment.

#### 2.3.2 Implementation details

All the neural network training works are implemented in PyTorch (Paszke et al., 2019). In terms of the registration, DiffusionNet is adapted from the open-source implementation (Sharp et al., 2022), including six blocks with 256 channels, and other settings follow the suggested value. First 48 eigenfunctions are incorporated in functional map inference. The network weights are updated with ADAM optimizer for 500 epochs and a batch size of 4. Please refer to (Tang et al., 2022) for more details.

SpiralNet++ is adopted as the VAE encoder backbone, configured with three spiral convolution layers. Each layer contains 128 channels, and a spiral length is 10. The weights are set to 0.9 for the reconstruction term and 0.1 for the divergence term in (10). Model training is performed using the Adam optimizer with an initial learning rate of 3 × 10^−4^, a batch size of 8, and for 800 training epochs. The results show that the model converged at approximately the 30th epoch.

The FEA simulations are carried out in LS-Dyna Software (ANSYS Inc., PA, US). The impactor goes 20 mm in 10 s towards the bone mid-shafts, where the proximal and distal ends are rotationally constrained through being partially rigidified while the distal end is allowed to translate longitudinally to formulate a pure three-point bending case.

## 3 Results

### 3.1 Registration of 3D non-rigid shapes

The self-supervised spectral contrastive learning embeds high-dimensional shape features, that encode multiscale intrinsic properties from per-vertex perspectives. Anatomically similar points are close in feature space regardless of the tessellation, and *vice versa*.

**FIGURE 4:**
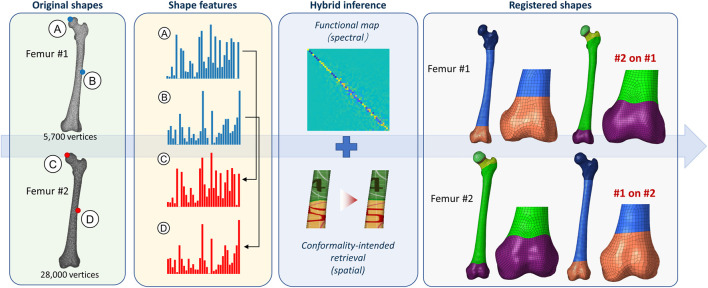
Self-supervised non-rigid registration. The original femur shapes #1 and #2 are fed into the pretrained DiffusionNet, achieving geometrically informative shape features that distinguish points with different anatomical meanings. Hybrid inference pipeline concatenating functional map and conformality-intended retrieval is applied to the learned features. High-fidelity pointwise correspondences are established finally, with smooth mesh pattern transfer that reveals the representability and inference robustness of the proposed registration approach. The registered instances have their shape and mesh swapped compared to the original. The “#1 on #2” denotes femur #1 mesh on femur #2 shape, and *vice versa*.

High-fidelity pointwise correspondences are inferred (Figure 4), wherein functional map and spectral-spatial optimizations are sequentially concatenated. Geometric adjacencies denoted in mesh pattern and textures are smoothly transferred between near-isometric femur shapes following the inference pipeline. The correspondences are intrinsic-approaching and conformal at vertices, i.e., incidental angles are preserved.

### 3.2 3D explicit/mesh generation

After the autoregressive pretraining, the generative VAE in Figure 3 encodes prior distribution of the collected femur shapes into a standard 6-dimensional Gaussian distribution. We can intuitively sample from the latent space and pass the sampled vector through the decoder to reconstruct an ordered vertex coordinate array, and synthesize new valid femur shapes by concatenating vertices with the inter-vertex connectivity on template.

**FIGURE 5:**
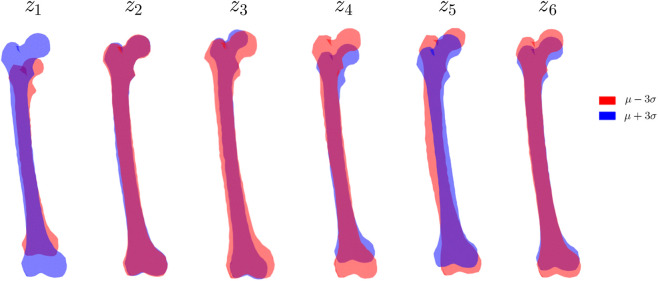
Influence of each individual VAE latent dimension on generated shapes. One dimension at a time of the latent vector is changed from its μ+3σ to μ−3σ (i.e., −3 to +3 since the latent space approximately follows a standard Gaussian distribution) with the other dimensions constantly held as μ (i.e., 0 for standard Gaussian).

It is practical to continuously control morphology of the synthesized femurs through tuning the latent vector (Figure 5), i.e., overall size ( $z_{1}$ ), fine-level shaft curvature ( $z_{2}$ ), slenderness ( $z_{3}$ ), shaft length ( $z_{4}$ ), overall bending curvature ( $z_{5}$ ), and local epiphysis/metaphysis style ( $z_{6}$ ). In terms of the first dimension ( $z_{1}$ ), from −3σ to +3σ , the decoded femur size is increased medial-laterally by 28.134 mm (a 31.33% fluctuation), anterior–posteriorly by 14.784 mm (22.18%), and longitudinally by 83.400 mm (23.33%). The fifth latent dimension ( $z_{5}$ ) controls the overall bending curvature of the femur. We select the mid-shaft section between the lesser trochanter and condyle areas and regress the centerline curvature. Results demonstrate that the average fitted curvature increased, while, conversely, the equivalent radius decreased significantly to between 66.80 mm and 862.57 mm. The third latent dimension ( $z_{3}$ ), from −3σ to +3σ , the decoded femur size is decreased medial-laterally by 21.940 mm (18.03%), and anterior–posteriorly by 14.813 mm (18.00%). The fourth latent dimension ( $z_{4}$ ), from −σ to +σ , the decoded femur size is decreased longitudinally by 65.679 mm (15.69%). The second latent dimension ( $z_{2}$ ), from −3σ to +3σ , the shaft gradually straightens.

The generated shapes are considerably fidelic with coherent geometric details and overall anatomical significance. The generation is even valid without unreal artefacts on the corner cases ( μ±3σ ), which implies that the latent space essentially encodes the prior distribution in a nearly diffeomorphic way.

### 3.3 From generation to simulation

**FIGURE 6:**
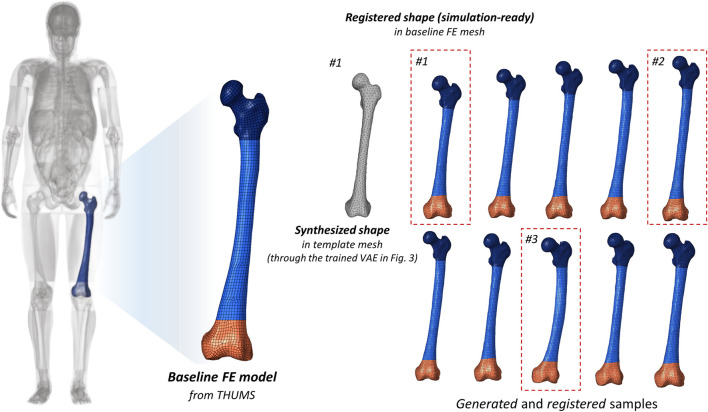
Simulation-ready samples in finite-element analysis format, through a joint “generation, registration and interpolation” pipeline.

In Figure 5, the synthesized shapes are subject to the template tessellations. We re-leverage non-rigid registration to map the baseline THUMS (Iwamoto et al., 2002) femur onto each generate shape respectively (Figure 6). The exteriors are registered and the interior nodes/elements are spatially interpolated through thin-plate spline (TPS) (Tang et al., 2020). Consequently, the synthesized femurs inherit the rigorous data structure and keywords from the baseline, differing only in their vertex coordinate contexts. Given this compatibility, and the fact that THUMS is an industry-standard model designed for vehicle safety R&D, we selected LS-Dyna as the prototype solver for validation.

Three representative samples with morphologically diverse features, e.g., length, slenderness, and shaft curvature, are selected. They are subject to three-point bending load in LS-Dyna. Overall, this setup replicates a simplified vehicle-to-pedestrian impact scenario, aligning with the design motivation and common application scenarios of LS-Dyna. Cylindrical impactors run into middle shafts along the lateral-medial trajectory, recapping a bonnet-to-thigh impact in a typical vehicle-to-pedestrian crash. The peak resultant cross-sectional moments at shaft area are 369, 168, and 423 Nm, respectively, with a significant difference (423 *versus* 168) due to the lower structural stiffness of the slender sample. The solvers founded on physical laws transfer the morphology inconsistency into diverse biomechanical responses, while the drastic discrepancy in structural reaction elucidates critical necessity to account for the morphological effect.

**FIGURE 7:**
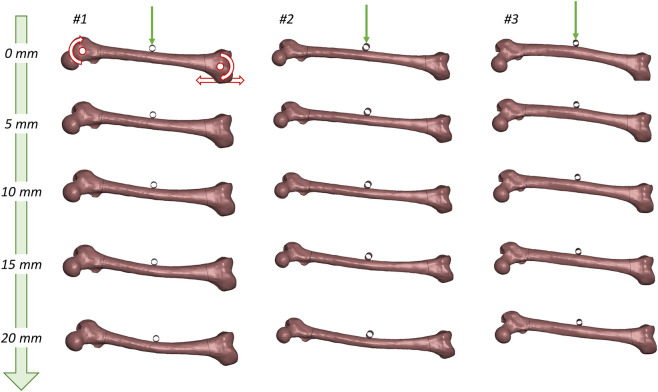
A production-level implementation demo. Three-point bending of sample #1, #2 and #3 in Figure 6, representing short, slender, and inversely curved femurs are simulated. Impactors transversely run into the middle shaft. The differences in bending patterns and resultant reactive moments (369, 168, and 423 Nm for #1 to #3) imply how population diversity in bone morphology brings about individual variation in biomechanics, and highlight the necessity of implementing personalized protection countermeasures to enhance traffic safety/impact protection on road. The practice essentially incorporates 3D generation and generative AI into industrial production.

The simulations in Figure 7 demonstrate that the proposed 3D explicit generative model straightforwardly benefits industrial practices, i.e., the model is production-ready. This is the principal objective of the current study. These demos serve to present the morphological diversity and numerical robustness of the synthesized shapes, verifying the entire conceptualization from shape generation to biomechanical analysis. Note that LS-Dyna and three-point bending are not the only applicable subjects; solvers like ANSYS and Abaqus, as well as other loading cases, are generally feasible under the proposed pipeline.

The generation (Figure 5), registration (Figure 6), and simulation (Figure 7) pipeline establishes a data-driven workflow that consolidates shape prior learning and high-fidelity map transfer to benefit finite-element modeling and analysis. This inspires us that developing a high-quality baseline model through high-fidelity case-by-case image reconstruction, i.e., reconstructing an anatomical human model (or some parts), and registering the baseline onto customized 3D AIGC (Artificial Intelligence Generated Content), might be a *time/cost-efficient, flexible, and controllable* way in next-generation study on biomechanics and other downstream tasks that are shape-sensitive.

## 4 Discussion

### 4.1 Case-by-case image reconstruction, SSM and generative artificial intelligence

This study demonstrates how generative artificial intelligence (GenAI) can fundamentally benefit and reshape diversified and individualized tissue modeling in biomechanical analysis. Conventional case-by-case image reconstruction heavily relies on medical images, and is not naturally generalizable. GenAI provides a revolutionary solution that learns the entire distribution and synthesize novel samples with very few marginal cost.

Compared to conventional SSMs based on PCA, the leveraged VAE architecture is better at capturing sophisticated distribution with irregular boundaries. PCA is essentially a linear dimensionality reduction algorithm, and the geometrically valid subset enclosed cannot be clearly distinguished. In contrast, VAE employs a deep neural network architecture to construct nonlinear encoder-decoder mappings and learn a smooth and compact latent manifold. That is, the original samples are nonlinearly “squeezed” into the latent. Figure 5 demonstrates that the VAE generates distinguishable and anatomically plausible shapes according to small latent changes. The generated shapes are continuous and free from visual or topological artifacts, indicating a smooth and coherent latent space that supports effective shape control and data augmentation. The variational nature of VAE prevents overfitting issues that are very popular in PCA analysis. Our experiments demonstrate that, within the context of this study, the VAE latent space is compact yet semantically explainable, that allows the users to deploy controllable generation in some practice.

Overall, this study demonstrates a closed pipeline that joins data-driven 3D synthesis and customized biomechanical analysis. It should be noted that the dynamic simulations performed in this work (Figure 7) serve primarily as a numerical verification of the synthetic shapes’ efficacy and robustness. Rather than aiming to provide an exhaustive biomechanical characterization of the femur, these cases qualitatively demonstrate the pipeline’s ability to capture and visualize morphology-induced variations in biomechanical responses. By ensuring that synthesized models are “simulation-ready” without manual intervention, this scheme addresses a major bottleneck in individualized modeling. Future tasks, e.g., injury mechanism study and protection countermeasure design/deployment strategy, can intuitively benefit from the proposed scheme.

### 4.2 Pros and Cons of explicit 3D generation

Implicit 3D generative models have dominated the field in the past few years (Liu et al., 2021; Kosiorek et al., 2021; Park et al., 2019) while implicit models synthesize astonishingly photorealistic scenes and images, the outputs cannot stably satisfy the quality requirements in engineering production. For example, finite-element analysis (Figure 7) as the most popular biomechanics investigation approach deals with beam/shell/solid elements rather than NeRF or SDF.

Mesh is natively compatible with industrial solvers and rendering engines in practice, and is locally controllable and editable. However, the highly discrete nature poses critical challenges in shape prior learning, as neural networks (NNs) cannot extract informative features from a group of chaotic tessellations. Thus one of motivations of the current study is to canonicalize the agnostic discretization and enable end-to-end shape prior learning on vertex coordinate space.

We leverage high-fidelity non-rigid registration to compile the underlying topology. This concept originates from our previous work, as introduced in Section 2.2.1. Non-rigid registration transforms the agnostic collections, i.e., the recruited 90 femurs, into globally aligned mesh topology, enabling autoregressive learning of the vertex distribution. Registration also facilitates smooth pattern transfer path between the baseline and the synthetic samples (Figures 4 and 6), that completes the entire “generation-simulation” workflow with shapes in explicit representation.

### 4.3 Towards 3D foundation model for generative tissue modeling

Modern generative AI studies highly advocates scaling raw, that “the more data consumed, the better the performance will be”. LLMs appreciate tokenization and autoregressive scheme (Radford et al., 2018; Bi et al., 2024; Team et al., 2025), which facilitates end-to-end, unsupervised pretraining. In the context of our study, we argue that 3D anatomical modeling is entering a similar phase. The field inevitably necessitates neural network architectures that can scale up accordingly which can consume a huge amount of imaging data for better performance. In this session, we discuss the roadmap to constructing general 3D foundation model. In terms of 3D foundation model, it refers to a universal generative framework trained on massive diverse anatomical datasets consolidating multiple components. The goal is to synthesize high-fidelity production-ready 3D structures that capture the full spectrum of human morphological variation. This is driven by the fact that the growth of radiological data is currently outpacing the processing capacity of classical processing algorithms.

The answer lies in annotation-efficiency and end-to-end training scheme. Our proposed end-to-end automated architecture is designed to bridge this gap enabling population-scale biomechanical analysis. We leverage registration to align the discretization-agnostic data structures, which is self-supervised and naturally annotation-efficient. The autoencoder nature of VAE makes the prior learning free of manual intervention as well, so we can seamlessly learn the shape prior from the group of recruited samples in an end-to-end manner.

### 4.4 Limitations and Prospects

As an essentially artificial intelligence study, there are a lot of tunable hyperparameters in the network design and training process and the incorporated models are updatable as well. Most basically, VAE as the generator is very classical. This study leverages VAE, but it is not a “must-be” choice. The KL-divergence in ELBO does not usually reconcile with the reconstructive loss. Consequently, VAEs suffer from “blurry” generations with poor fidelity. Diffusion models, e.g., DDPM (Ho et al., 2020), learn to iteratively denoise the perturbed samples and lead to a more harmonic transport between Gaussian latent and the prior distribution, so they might be a better choice for future generator design. The reconstructive loss *L*_1_ distance, i.e., the Euclidean 1-norm of the vertex distances, intuitively aligns the targets, but the gradient flow is unaware of how the vertex of interest jointed with the neighborhood. Geometric metrics on manifold deflections, e.g., Chamfer distance, might be beneficial in this regard as they are structurally informed.

While our framework demonstrates robust performance, we acknowledge several theoretical and practical scenarios where it may encounter difficulties. First, our registration component is primarily intrinsic and well-suited for near-isometric perturbations. Therefore, tissues that undergo extreme large-scale deformations or topological shifts such as certain soft tissues like the female breast or highly mobile internal organs may pose a challenge. In these cases, the assumption of metric stability may no longer hold leading to potential inaccuracies in correspondence. Second, our model utilizes DiffusionNet for feature aggregation within the tangent space. This approach might struggle with sharp geometries like the vertebrae which possess numerous sharp edges and high-curvature regions as they present challenges for stable tangent space estimation and smooth feature propagation. Furthermore, multi-scale complexity poses another hurdle. When modeling the entire human body, the model must simultaneously account for large-scale torso geometry and fine-scale details like fingers. The drastic variance in feature resolution between these regions can lead to incompatibility of metric-relied message propagation. Finally, our model currently operates from a purely geometric perspective and does not explicitly decouple shape from pose. Learning the complex non-linear coupling between anatomical variation and functional posture remains an ongoing challenge that we intend to address in future research.

The current study works only with femur shape. The proposed registration and generation paradigms are theoretically scalable, so we expect to incorporate more body segments in near future and formulate a larger-scale model to synthesize multiple anatomical structures simultaneously. Besides, performance of the generated samples in downstream productions, e.g., rendering, physical simulation, is to be emphasized. Standard, domain-specific benchmarks are of profound significance to regularize the model developments and promote industry-level productions.

The presented scheme is a visionary prototype that depicts the data-driven workflow for biomechanical analysis. It is simple as a preliminary demo but is complete with all fundamental functional modules. In future, we look forward to synthesizing and incorporating more samples and delivering a drastically larger matrix to sufficiently investigate morphological effect on human biomechanics.

## 5 Conclusion

This work introduces a data-driven framework that leverages generative artificial intelligence to revolutionize the tissue modeling pipeline in biomechanical analysis. We have tackled several bottlenecks that prohibit 3D generation with explicit presentations through consolidating geometric deep learning and discrete differential geometry. We propose to learn geometry-informed features through self-supervised pretraining on spectral domain, enhance conventional functional map with conformality-intended optimization, and infer high-fidelity correspondences accordingly in a hybrid spectral-spatial manner. A variational autoencoder (VAE) with 6-dimensional latent space is trained on a group of 90 recruited, structurally aligned femur shapes. The latent dimensions control different morphological patterns, i.e., overall size, overall bending curvature, slenderness, shaft length, fine-level shaft curvature, and local epiphysis/metaphysis style. The synthesized samples vary with both shape and dimensions, e.g., tuning the first latent results in an 83.4 mm change in femur length. The VAE model can synthesize geometrically valid shapes within up to 3 standard deviations (>99.7%), advocating strong compactness of representability of the learned prior. 10 new femurs are synthesized and registered onto a baseline finite-element model, with each processed in less than 100 s. Preliminary analysis with three-point bending load in LS-Dyna reveals the morphology variation might lead to drastically distinct deformation responses, and can bring about significant difference in bending biomechanics.

This study establishes a contemporary generative paradigm for tissue modeling, and demonstrates the efficacy and feasibility of bridging source data and production, i.e., medical images and biomechanical analysis. Our method is time-efficient, productive, and even creative. The pretraining scheme is promisingly scalable and might inspire 3D foundation model. The presented scheme is a visionary prototype, and will significantly benefit digital twin analysis in various morphology/biomechanics-centered tasks.

## Data Availability

The datasets presented in this article are not readily available because they are subject to ethical restrictions regarding participant privacy and are managed under a data-sharing agreement with third-party collaborators. Requests to access the datasets should be directed to Dr. Tang, Jisi (drjisitang@gmail.com).

## References

[B1] Bi D.-A. X. Chen D. Chen G. Chen S. Dai D. Deng C. (2024). DeepSeek LLM: scaling open-source language models with longtermism. ArXiv 2024;abs/2401.02954.

[B2] Carter P. M. Flannagan C. A. C. Reed M. P. Cunningham R. M. Rupp J. D. (2014). Comparing the effects of age, BMI and gender on severe injury (AIS 3+) in motor-vehicle crashes. Accid. Analysis & Prev. 72, 146–160. 10.1016/j.aap.2014.05.024 25061920 PMC4753843

[B3] Çiçek Ö. Abdulkadir A. Lienkamp S. S. Brox T. Ronneberger O. (2016). “3D U-Net: learning dense volumetric segmentation from sparse annotation,” International conference on medical image computing and computer-assisted intervention (Springer).

[B4] Daniel S. Mario B. (2019). The polygon mesh processing Library. Available online at: http://www.pmp-library.org (Accessed March 18, 2026).

[B5] Du W. Zhang J. Hu J. (2018). “A method to determine cortical bone thickness of human femur and tibia using clinical CT scans,” 2018 IRCOBI conference proceedings, Athens (Greece).

[B6] Ezuz D. Ben-Chen M. (2017). Deblurring and denoising of maps between shapes. Computer graphics forum (Wiley Online Library).

[B7] Gong S. Chen L. Bronstein M. Zafeiriou S. (2019). “Spiralnet++: a fast and highly efficient mesh convolution operator,” Proceedings of the IEEE/CVF international conference on computer vision workshops.

[B8] Goodfellow I. Pouget-Abadie J. Mirza M. Xu B. Warde-Farley D. Ozair S. (2020). Generative adversarial networks. Commun. ACM 63 (11), 139–144. 10.1145/3422622

[B9] Hayashi S. Yasuki T. Kitagawa Y. (2008). Occupant kinematics and estimated effectiveness of side airbags in pole side impacts using a human FE model with internal organs. SAE Tech. Pap. 10.4271/2008-22-0015 19085170

[B10] Higgins I. Matthey L. Pal A. Burgess C. Glorot X. Botvinick M. (2017). beta-vae: learning basic visual concepts with a constrained variational framework. International conference on learning representations.

[B11] Ho J. Jain A. Abbeel P. (2020). Denoising diffusion probabilistic models. Adv. Neural Inf. Process. Syst. 33, 6840–6851. 10.48550/arXiv.2006.11239

[B12] Hu J. Zhang K. Reed M. P. Wang J.-T. Neal M. Lin C.-H. (2019). Frontal crash simulations using parametric human models representing a diverse population. Traffic Inj. Prev. 20 (Suppl. 1), S97–S105. 10.1080/15389588.2019.1581926 31381451

[B13] Iwamoto M. Kisanuki Y. Watanabe I. Furusu K. Miki K. Hasegawa J. (2002). “Development of a finite element model of the total human model for safety (THUMS) and application to injury reconstruction,” Proceedings of the international IRCOBI conference.

[B14] Johnson J. Ravi N. Reizenstein J. Novotny D. Tulsiani S. Lassner C. (2020).“Accelerating 3d deep learning with pytorch3d,” in SIGGRAPH Asia 2020 courses, 1.

[B15] Kerbl B. Kopanas G. Leimkühler T. Drettakis G. (2023). 3D Gaussian splatting for real-time radiance field rendering. ACM Trans. Graph 42 (4), 139:1–14. 10.1145/3592433

[B16] Kingma D. P. Welling M. (2013). Auto-encoding variational bayes. arXiv arXiv:13126114 10.48550/arXiv.1312.6114

[B17] Klein K. F. Hu J. Reed M. P. Hoff C. N. Rupp J. D. (2015). Development and validation of statistical models of femur geometry for use with parametric finite element models. Ann. Biomed. Eng. 43 (10), 2503–2514. 10.1007/s10439-015-1307-6 25808208

[B18] Kosiorek A. R. Strathmann H. Zoran D. Moreno P. Schneider R. Mokr'a S. (2021). NeRF-VAE: a geometry aware 3D scene generative model. ArXiv., 00587. abs/2104. 10.48550/arXiv.2104.00587

[B19] Liu S. Zhang X. Zhang Z. Zhang R. Zhu J.-Y. Russell B. C. (2021). Editing conditional radiance fields,” in 2021 IEEE/CVF international conference on computer vision (ICCV), 5753–5763.

[B20] Mildenhall B. Srinivasan P. P. Tancik M. Barron J. T. Ramamoorthi R. Ng R. (2021). Nerf: representing scenes as neural radiance fields for view synthesis. Commun. ACM 65 (1), 99–106. 10.1145/3503250

[B21] Ovsjanikov M. Ben-Chen M. Solomon J. Butscher A. Guibas L. (2012). Functional maps: a flexible representation of maps between shapes. ACM Trans. Graph. (ToG) 31 (4), 1–11. 10.1145/2185520.2185526

[B22] Park J. J. Florence P. R. Straub J. Newcombe R. A. Lovegrove S. (2019). DeepSDF: learning continuous signed distance functions for shape representation,” in 2019 IEEE/CVF conference on computer vision and pattern recognition (CVPR), 165–174.

[B23] Paszke A. Gross S. Massa F. Lerer A. Bradbury J. Chanan G. (2019). Pytorch: an imperative style, high-performance deep learning library. Adv. Neural Inf. Process. Syst. 32, 8024–8035. 10.48550/arXiv.1912.01703

[B24] Radford A. Narasimhan K. Salimans T. Sutskever I. (2018). Improving language understanding by generative pre-training.

[B25] Sharp N. Attaiki S. Crane K. Ovsjanikov M. (2022). Diffusionnet: discretization agnostic learning on surfaces. ACM Trans. Graph. (TOG) 41 (3), 1–16. 10.1145/3507905

[B26] Shi X. Cao L. Reed M. P. Rupp J. D. Hoff C. N. Hu J. (2014). A statistical human rib cage geometry model accounting for variations by age, sex, stature and body mass index. J. Biomech. 47 (10), 2277–2285. 10.1016/j.jbiomech.2014.04.045 24861634

[B27] Sohl-Dickstein J. Weiss E. Maheswaranathan N. Ganguli S. (2015). “Deep unsupervised learning using nonequilibrium thermodynamics”, in International conference on machine learning. (Proceedings of Machine Learning Research).

[B28] Tang J. Zhou Q. Nie B. Hu J. (2020). Obesity effects on pedestrian lower extremity injuries in vehicle-to-pedestrian impacts: a numerical investigation using human body models. Traffic Inj. Prev. 21 (8), 569–574. 10.1080/15389588.2020.1821195 33095068

[B29] Tang J. Zhou Q. Huang Y. (2022). Self-supervised learning for non-rigid registration between near-isometric 3D surfaces in medical imaging. IEEE Trans. Med. Imaging 42 (2), 519–532. 10.1109/TMI.2022.3218662 36318555

[B30] Team K. Du A. Gao B. Xing B. Jiang C. Chen C. (2025). Kimi k1.5: scaling reinforcement learning with LLMs. ArXiv 2025;abs/2501.12599

[B31] Zhang K. Cao L. Fanta A. Reed M. P. Neal M. Wang J.-T. (2017). An automated method to morph finite element whole-body human models with a wide range of stature and body shape for both men and women. J. Biomech. 60, 253–260. 10.1016/j.jbiomech.2017.06.015 28668185

